# Photosynthetic Conversion of CO_2_ Into Pinene Using Engineered *Synechococcus* sp. PCC 7002

**DOI:** 10.3389/fbioe.2021.779437

**Published:** 2021-12-17

**Authors:** Ruigang Yang, Lingyun Zhu, Tao Li, Lv-yun Zhu, Zi Ye, Dongyi Zhang

**Affiliations:** ^1^ Department of Biology and Chemistry, College of Liberal Arts and Sciences, National University of Defense Technology, Changsha, China; ^2^ State Key Laboratory of Freshwater Ecology and Biotechnology, Institute of Hydrobiology, Chinese Academy of Sciences, Wuhan, China; ^3^ Hunan Key Laboratory of Economic Crops, Genetic Improvement, and Integrated Utilization, School of Life Sciences, Hunan University of Science and Technology, Xiangtan, China

**Keywords:** terpenoids, pinene, cyanobacteria, pinene synthase, dodecane

## Abstract

Metabolic engineering of cyanobacteria has received much attention as a sustainable strategy to convert CO_2_ to various longer carbon chain fuels. Pinene has become increasingly attractive since pinene dimers contain high volumetric energy and have been proposed to act as potential aircraft fuels. However, cyanobacteria cannot directly convert geranyl pyrophosphate into pinene due to the lack of endogenous pinene synthase. Herein, we integrated the gene encoding *Abies grandis* pinene synthase into the model cyanobacterium *Synechococcus* sp. PCC 7002 through homologous recombination. The genetically modified cyanobacteria achieved a pinene titer of 1.525 ± 0.l45 mg L^−1^ in the lab-scale tube photobioreactor with CO_2_ aeration. Specifically, the results showed a mixture of *α*- and *β*-pinene (∼33:67 ratio). The ratio of *β*-pinene in the product was significantly increased compared with that previously reported in the engineered *Escherichia coli*. Furthermore, we investigated the photoautotrophic growth performances of *Synechococcus* overlaid with different concentrations of dodecane. The work demonstrates that the engineered *Synechococcus* is a suitable potential platform for *β*-pinene production.

## Introduction

Growing concerns about environmental pollution issues and demands for alternative energy have stimulated photosynthetic organisms emerging as powerful platforms converting CO_2_ to various bio-based products. As one of the oldest living phyla (Schopf and Packer, 1987), cyanobacteria are oxygenic photosynthetic autotrophic bacteria widely distributed in aquatic and terrestrial habitats (Díez and Ininbergs, 2014). They play a crucial role in the biogeochemical cycles of carbon and nitrogen with the capacity of fixing carbon dioxide and atmospheric nitrogen (Parmar et al., 2011). Moreover, cyanobacteria possess the dramatic property of photosynthetically converting CO_2_ to various beneficial organic compounds, including drugs (Simmons et al., 2005; Teruya et al., 2009), cosmetics (Conde et al., 2000; Rajneesh et al., 2017), food additives (Ma et al., 2019), and biofuels (Sarma et al., 2016; Singh et al., 2016; Choi et al., 2020), benefiting from high growth rates, ease of genetic engineering, and large-scale cultivation (Rajneesh et al., 2017). In addition, the increasingly abounding toolboxes for genetic manipulation impel cyanobacteria to become attractive platforms for bio-based production (Markley et al., 2015; Carroll et al., 2018; Sun et al., 2018; Xia et al., 2019).

Terpenoids are a large group of high-value compounds and have been successfully produced by multiple microbial cell factories (Davies et al., 2015; Wang et al., 2018; Moser and Pichler, 2019). Cyanobacteria synthesize terpenoid precursors isopentenyl diphosphate (IPP) and dimethylallyl diphosphate (DMAPP) with the endogenous methylerythritol phosphate (MEP) pathway (Lin and Pakrasi, 2019), which has been engineered to produce various terpenoids (Pattanaik and Lindberg, 2015; Betterle and Melis, 2019). Pinene is a plant natural monoterpene (C10) and has shown a wide range of commercial applications in flavorings (Gomes-Carneiro et al., 2005), fragrances (Kirby and Keasling, 2009), and pharmaceuticals (Zhao et al., 2018). Furthermore, pinene dimers’ high energy density is comparable to tactical fuels JP-10, and this has made pinene emerge as a potential bio-jet fuel used in aircraft and aircraft-launched missiles (Harvey et al., 2010).

Microbial factories have been employed to produce pinene with various genetic tools. Currently, the highest pinene production efficiency was achieved in the engineered *Escherichia coli* (*E. coli*) and yeast, reaching respective titers of 166.5 mg L^−1^ (Niu et al., 2018) and 36.1 mg L^−1^ (Wei et al., 2021). In order to build a photosynthetic microbial platform for pinene production, several efforts have been successfully carried in the photosynthetic microorganisms, including *Rhodobacter sphaeroides* and *Synechocystis* sp. PCC 6803 (Tashiro et al., 2016; Wu et al., 2021). The highest pinene production titer was achieved at 0.540 mg L^−1^ in the engineered *R. sphaeroides* by optimizing the expression of the critical enzymes in the MEP pathway (Wu et al., 2021). In contrast, only 0.08 mg L^−1^ pinene was harvested with the cold trap in the transgenic *Synechocystis* sp. PCC 6803 integrated with the mutant pinene synthase (PS) gene (Tashiro et al., 2016). Developing a superior cyanobacterial chassis for synthetic biology and metabolic engineering applications is supposed to be an effective effort to further improve the productivities of terpenoids for commercial production.

In cyanobacteria, the condensation reaction of IPP and DMAPP catalyzed by geranyl diphosphate synthase (GPPS) produces geranyl diphosphate (GPP), the substrate of PS. In the present study, we engineered *Synechococcus* sp. PCC 7002 (*Synechococcus*) to produce pinene by introducing a recombinant plasmid containing the *Abies grandis* PS (AgPS) gene. The AgPS gene was integrated into the cyanobacteria genome by homologous recombination and was overexpressed under the control of the *cpcBA* (phycocyanin) operon promoter (*PcpcBA*) from *Synechocystis* sp. PCC 6803 (Xu et al., 2011). The successful pinene production of transgenic *Synechococcus* was achieved at the highest rate of 1.525 ± 0.l45 mg L^−1^ under the condition of CO_2_ aeration and continuous light. Furthermore, the ratio of two pinene isomers, *α*- and *β*-pinene, was also determined in the final product.

## Materials and Methods

### Strains and Construction of Plasmids

Strains and plasmids used in this study are listed in Table 1. The AgPS gene was amplified from pAgGPPS-(GSG)_2_-AgPS by PCR with specific primers AgPS_F: 5′-CAG​CAT​ATG​CGT​CGT​G-GTA​AAT​CTA​TCA​C-3′ and AgPS_R: 5′-GCG​GGA​TCC​TTA-CAG​CGG​AAC​AGA​TTC​CAG-3′. The PCR product was purified and ligated into the cloning vector pBZ (TransGen Biotech, China) to perform sequencing. The AgPS gene with the correct sequence was inserted into pAQ1EX-PcpcBA to generate pAQ1EX-AgPS plasmid *via Nde*I and *Bam*HI (Thermo Scientific) digestion. The plasmid pAQ1EX-AgPS was aiming at integrating the functional genes into the endogenous high-copy plasmid pAQ1 through homologous recombination. A modified protocol following that described previously (Stevens and Porter, 1980; Frigaard et al., 2004) was used to transform pAQ1EX-AgPS into *Synechococcus*. To select the transformant strain, 100 μg ml^−1^ streptomycin was applied, and the successful transgene incorporation was confirmed by colony PCR and sequencing using the primers PAQIN_F: 5′-GGA​ATT​GTG​CGT​GTG​GTT​TC-3′ and PAQIN_R: 5′-CTA​ACG​ATC​AGC​GCG​AAA​AG-3′.

**TABLE 1:** Strains and plasmids used in this study.

Name	Description	Source
Strains
*Escherichia coli* Trelief™ 5*α*	*E. coli* strain used for cloning and plasmid construction	Tsingke, China
WT	Model marine cyanobacterium *Synechococcus* sp. PCC 7002 used as the host for transgene integration	Yang et al. (2013)
7002-PS	*Synechococcus* with streptomycin resistance (Sm^R^) cassette and AgPS gene	This work
Plasmids
pBZ	pEASY®-Blunt Zero Cloning Vector containing a suicide gene mutated by ligation of PCR fragment	TransGen Biotech, China
pAQ1EX	*Synechococcus* expression vector containing flanking regions of endogenous plasmid pAQ1 in *Synechococcus* for homologous recombination of Sm^R^ cassette and the integrated gene driven by *PcpcBA*	Xu et al. (2011)
pAgGPPS-(GSG)_2_-AgPS	Plasmid harboring codon-optimized fusion genes of *A. grandis* GPPS and AgPS with a (GSG)_2_ linker	Sarria et al. (2014)
pBS-AgPS	pBZ harboring AgPS gene for cloning and sequencing	This work
pAQ1EX-AgPS	pAQ1EX harboring AgPS gene and Sm^R^ cassette	This work

### Growth Experiments

*Synechococcus* wild-type (WT) and AgPS transformant (7002-PS) strains were grown in liquid medium A+ containing the following: 18 g L^−1^ NaCl, 5 g L^−1^ MgSO_4_ 7H_2_O, 1 g L^−1^ NaNO_3_, 0.6 g L^−1^ KCl, 0.050 g L^−1^ KH_2_PO_4_, 0.270 g L^−1^ CaCl_2_, 0.020 g L^−1^ Na_2_CO_3_, 0.030 g L^−1^ Na_2_EDTA·2H_2_O, 3.890 mg L^−1^ FeCl_3_·6H_2_O, 1 g L^−1^ Tris HCl (pH 8.2), 2.860 mg L^−1^ H_3_BO_3_, 1.810 mg L^−1^ MnCl_2_·4H_2_O, 0.220 mg L^−1^ ZnSO_4_·7H_2_O, 0.390 mg L^−1^ Na_2_MoO_4_·2H_2_O, 0.100 mg L^−1^ CuSO_4_·5H_2_O, 0.050 mg L^−1^ Co(NO_3_)_2_·6H_2_O, and 4 μg L^−1^ vitamin B12 (Stevens and Porter, 1980; Ludwig and Bryant, 2011). The 7002-PS strain containing Sm^R^ cassette was selected on solid A+ medium, adding 1.2% (*w*/*v*) Bacto Agar (BD) and streptomycin with the required concentration. Seed cultures were performed in 50-ml Erlenmeyer flasks using an orbital shaker at 100 r min^−1^. The flasks contained 30 ml of liquid A+ medium in the presence of antibiotic. All *Synechococcus* strains were grown at 30°C under continuous illumination of 100 μmol photons m^−2^ s^−1^, and optical density (OD) was measured to monitor cell growth using a spectrophotometer (Varian) at 730 nm.

### Western Blotting

*Synechococcus* cultures used for western blotting were inoculated into 50 ml fresh A+ medium with an initial OD_730_ = 0.1 and grown for 24 h under continuous aeration supplement with 1% (*v*/*v*) CO_2_. Cells were harvested by centrifugation and disrupted with a motor-driven tissue grinder (Sangon Biotech). Equal amounts of total proteins in crude cell lysates were subjected to western blotting analysis. AgPS was identified with anti-His mouse monoclonal antibody (TransGen Biotech, China) followed by goat anti-mouse IgG (H + L) (TransGen Biotech, China) and visualized using a DAB horseradish peroxidase color development kit (Beyotime, China).

### Pulse Amplitude Modulation Fluorometry

The Water-PAM (Walz) was used to measure the variable chlorophyll fluorescence with a Water-S stirring device capable of keeping the samples homogenous and preventing sedimentation of the cells. Light-emitting diodes supplied red measuring light (spectral peak at 650 nm), actinic light, and saturation pulses (spectral peak at 660 nm). After 20 min of dark adaption, a 2-ml sample was used to analyze fluorescence parameters Fv/Fm by measuring Fo and Fm. Light treatment strategy and stirrer operation in this study were according to the previous reports (Campbell et al., 1998; Cosgrove and Borowitzka, 2006).

### Pinene Production and Analysis

*Synechococcus* cultures used for pinene production were inoculated into 60 ml fresh A+ medium without antibiotic with an initial OD_730_ = 1.0. The cultures were grown for 72 h under continuous aeration supplement with 1% (*v*/*v*) CO_2_, and a 6-ml dodecane (Aladdin) overlay was applied at the beginning to trap the pinene excreted from the cells. Samples of 500 μl dodecane overlay were harvested at the end of the production period. The procedure of sample pretreatment was following a modified method as described previously (Sarria et al., 2014), adding (R)-(+)-limonene (Aladdin) as an internal standard. These samples were analyzed on a gas chromatography–mass spectrometer (GC-MS) (Agilent 7890A with Agilent 5975C MS detector) by a standard curve of (−)-*α*- and *β*-pinene (Aladdin). The GC-MS was equipped with a DB-5MS column (30 m × 0.25 mm × 0.25 µm) to separate hydrophobic molecules. The analysis conditions were as follows: He (1 ml min^−1^) as a carrier gas, split ratio of 5:1; an injector temperature of 300°C; and an oven program of 50°C for 5 min, ramp at 10°C min^−1^ to 150°C, ramp at 30°C min^−1^ to 280°C and held for 5 min.

## Results and Discussion

### Determination of Candidate PS Genes

*Synechococcus* is capable of utilizing the endogenous MEP pathway to synthesize GPP. In order to build *Synechococcus* a platform for pinene production, we need to integrate an exogenous pinene synthase into the genome of *Synechococcus* to convert GPP into *α*- or *β*-pinene with high fidelity. Although both pinene isomers can be found in turpentine (Behr and Johnen, 2009), the *β*-pinene isomer is considered preferable for higher economic value (Sarria et al., 2014) and dimerization efficiency (Walls and Rios-Solis, 2020). Thus, the AgPS gene was selected to produce pinene with more proportion of *β*-pinene (Bohlmann et al., 1997) and had shown the highest activity for pinene synthesis in *E. coli* among the high-fidelity pinene synthases (Sarria et al., 2014).

### Construction of Synechococcus Strain Integrated With AgPS Transgene

**FIGURE 1:**
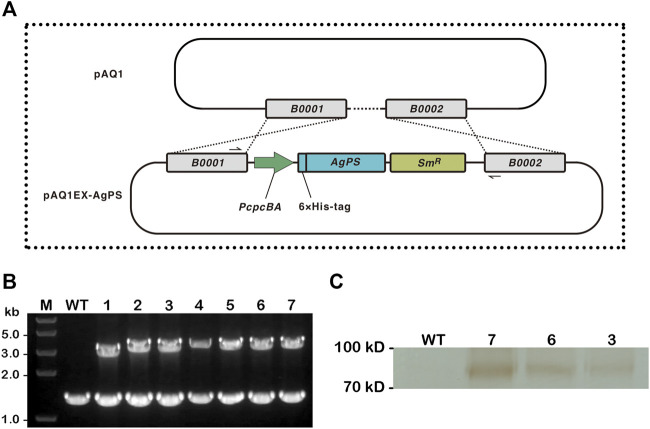
Construction of *Synechococcus* sp. PCC 7002 overexpressing *Abies grandis* pinene synthase. **(A)** Illustrations of the integration of endogenous plasmid pAQ1 in wild-type *Synechococcus* with the inserted *A. grandis* pinene synthase (AgPS) cassette in the recombinant plasmid pAQ1EX-AgPS. The AgPS cassette contained *PcpcBA* to drive transgene expression, and a spectinomycin resistance (Sm^R^) selectable marker. The specific primers PAQIN_F and PAQIN_R shown in black solid half arrows are used for demonstrating the successful integration of AgPS cassette into pAQ1 (between open-reading frames *B0001* and *B0002*) *via* double homologous recombination. **(B)** Confirmation of transgene integration by colony PCR using the above-mentioned primers. The wild-type (WT) strain and seven independent transformants of the 7002-PS strain (1–7) were verified. Lane M: *Trans*2K^®^ Plus II DNA Marker (TransGen Biotech, China). Each number above the image of the agarose gel correlated with PCR product. **(C)** Western blotting analysis of the total protein extract isolated from  WT and three independent transformants of the 7002-PS strain (3, 6, and 7). Sample from the WT strain was used as a control. Proteins were detected using anti-His mouse monoclonal antibody (TransGen Biotech, China) followed by the incubation with anti-mouse secondary antibody conjugated with HRP. Reactions were visualized using a DAB horseradish peroxidase color development kit (Beyotime, China).

The nucleotide sequence of the AgPS gene from Sarria et al. (2014) had been modified and expressed according to the codon usage preference of *E. coli*. A comparison of the modified AgPS gene sequence with the codon usage of *Synechococcus* was predicted with the Graphical Codon Usage Analyser (Fuhrmann et al., 2004), and the results showed that the relative adaptiveness values are all higher than 30% (**Supplementary Material S1**). We therefore directly amplified the modified AgPS gene from plasmid pAgGPPS-(GSG)_2_-AgPS and therewith integrated it into the pAQ1EX vector between the *Nde*I and *Bam*HI sites. The insertion of AgPS gene into the neutral region of pAQ1 between two open-reading frames encoding hypothetical proteins (Figure 1A) was confirmed by colony PCR and sequencing in numerous transformants of the 7002-PS strain (Figure 1B). Interestingly, although the 7002-PS strain was isolated using the streak plate method three times, as the colony PCR results showed, the neutral region of pAQ1 between two open-reading frames had still not been entirely replaced by the integrated sequence. We supposed that the region replaced by the transgenes in pAQ1EX-PS was essential for the growth of *Synechococcus* (Xu et al., 2011), and the complete integration of the exogenous genes in pAQ1EX was hard to realize. The potential different amount of the transgenic pAQ1 might eventually lead to the differences in pinene production performance among the independent transformants of the 7002-PS strain. The expression of AgPS was confirmed by western blotting (Figure 1C).

### The Effect of Dodecane on the Photoautotrophic Growth of *Synechococcus*

Dodecane overlay has been turned out as an effective method for terpenes harvesting (Davies et al., 2014; Sarria et al., 2014; Niu et al., 2018; Dienst et al., 2020). This article utilized 1% (*v*/*v*) CO_2_ bubbles to supply inorganic carbon for pinene production in the 7002-PS strain. Photoautotrophic cultivation of cyanobacteria with sodium bicarbonate as an inorganic carbon source and using orbital shakers or magnetic stirrers seem adequate for terpenes production (Silva et al., 2016). However, CO_2_ used as the inorganic carbon source has environmentally friendly advantages in integrating atmospheric CO_2_ into biomass. Although filling the upper space of the medium with CO_2_ could maintain continuous production of terpene (Davies et al., 2014), we need to develop deeper insight into the production performance of engineered strains grown with CO_2_ bubble aeration.

**FIGURE 2:**
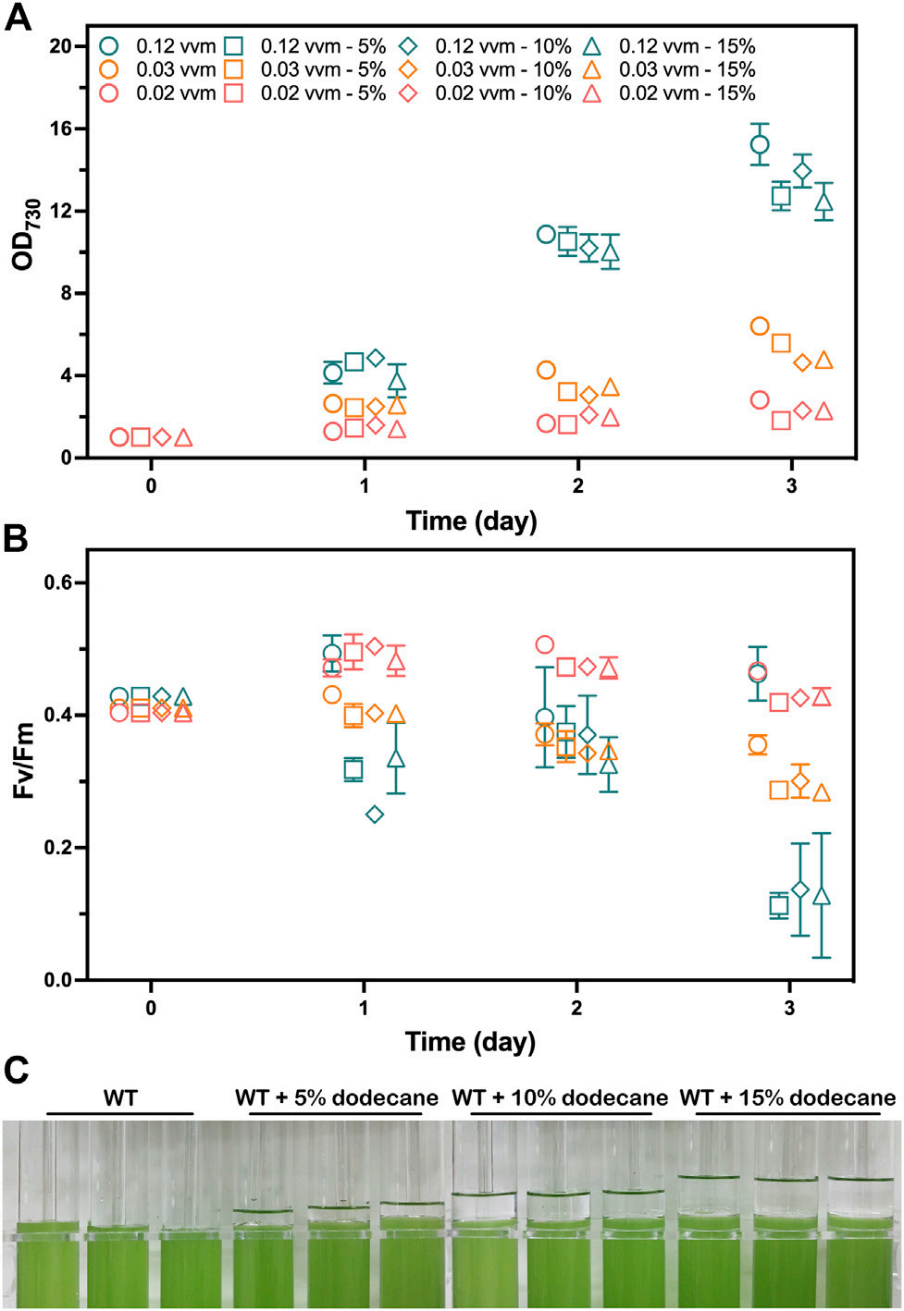
Photoautotrophic performance analysis of the WT strain grown with 1% (*v*/*v*) CO_2_ aeration at 0.12, 0.03, and 0.02 vvm for 3 days. The mediums were overlaid with 0%, 5%, 10%, and 15% (*v*/*v*) of dodecane, respectively. Samples were collected for the analysis at 1-day intervals. **(A)** The photoautotrophic growth parameters monitored by OD_730_. **(B)** Photosynthetic efficiency evaluated by chlorophyll fluorescence parameter Fv/Fm. The works on the WT strain grown with 0%, 5%, 10%, and 15% (*v*/*v*) of dodecane overlay are shown as circles, squares, diamonds, and triangles, respectively. The works on the WT strain grown with 1% (*v*/*v*) CO_2_ aeration at 0.12, 0.03, and 0.02 vvm are shown as teal, tangerine, and salmon colors, respectively. **(C)** Image of the WT strain cultivated in A+ medium with 0%, 5%, 10%, and 15% (*v*/*v*) of dedocane overlay, respectively. The error bars represent standard deviations of means (mean ± SD, *n* = 3).

To investigate whether dodecane overlay limits the photoautotrophic growth of cyanobacteria ventilated with CO_2_, we inspected the photoautotrophic growth of the WT strain overlaid with different dodecane concentrations over a 72-h time course at various aeration rates. Cell growth rates were measured by OD_730,_ and the photosynthetic performance was evaluated by the chlorophyll fluorescence parameter Fv/Fm. The chlorophyll fluorescence parameter Fv/Fm and OD_730_ of the WT cells were compared when growing with 0%, 5%, 10%, and 15% (*v*/*v*) dodecane overlay. As the organic phase could not maintain stability with an aeration rate higher than 0.12 vvm (volume of gas per volume of liquid per minute) in this work, the aeration rates were initially set to 0.02, 0.03, and 0.12 vvm. Apparently, the limited CO_2_ provision caused by the lower aeration rates (0.02 and 0.03 vvm) had an adverse effect on the photoautotrophic growth of *Synechococcus*. Meanwhile, the OD_730_ of the WT cells cultivated with aeration rate at 0.12 vvm exhibited obvious variation (Figure 2A). The chlorophyll fluorescence parameter Fv/Fm of the cells without dodecane overlay was higher than the that of the cells overlaid with dodecane after 2 days, and the cells aerating at 0.12 vvm presented the greatest gap, which was supposed to aggravate the difference of OD_730_ (Figure 2B). However, neither the Fv/Fm nor OD_730_ of the WT cells showed apparent difference whenever the aeration rate was 0.02 or 0.03 vvm. The results indicated that the dodecane overlay led to a slight restriction to the photosynthetic growth of *Synechococcus* cultivated with CO_2_ bubble aeration at a high rate (0.12 vvm), and the three concentrations (5%, 10%, and 15%) of the dodecane overlay had no significant difference in the limitation effect. However, the dramatic variance of Fv/Fm between the WT cells overlaid with and without dodecane demonstrated that cultivation with dodecane overlay at a high aeration rate enables to impair the photosystem II efficiency of *Synechococcus*, which suggests that dodecane overlay is not the best choice for long-term product harvest in the condition of high gas aeration rate for *Synechococcus*.

The different concentrations of dodecane overlay resulted in the liquid level differences of the organic phase in the tube photobioreactor (Figure 2C). Considering the disturbance of the interface between two phases caused by bubble breakage, the moderate thickness of the organic phase is supposed to be of great significance for reducing pinene loss and the inhibition effect of dodecane overlay on cell growth. Besides, a slight loss of dodecane volume caused by gas aeration was observed on the tube wall (data not shown), which demanded an appropriate volume to reduce the calculation error caused by the volume change. Thus, we determined that a 10% (*v*/*v*) dodecane overlay was suitable for pinene harvesting from the culture medium in our tube photobioreactor.

### Growth and Pinene Production of the 7002-PS Strain

**FIGURE 3:**
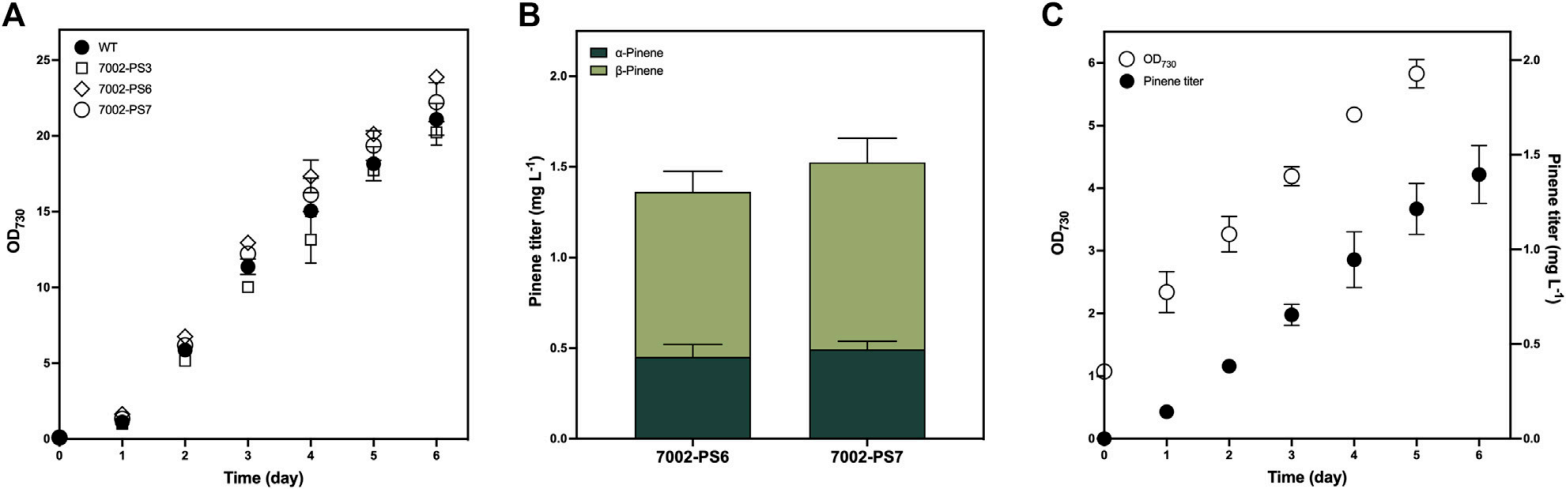
Growth and pinene production of the 7002-PS strain. **(A)** Photoautotrophic growth of the WT strain and three independent transformants of the 7002-PS strain grown with 1% (*v*/*v*) CO_2_ aeration at 0.12 vvm for 6 days. Samples were collected for the analysis at 1-day intervals. **(B)** Pinene production of 7002-PS6 and 7002-PS7 after a 72-h cultivation with 10% (*v*/*v*) dodecane overlay and 1% (*v*/*v*) CO_2_ aeration at 0.12 vvm. **(C)** Photoautotrophic growth and pinene production of 7002-PS7 grown with 10% (*v*/*v*) dodecane overlay and 1% (*v*/*v*) CO_2_ aeration at 0.03 vvm for 6 days. Samples were collected for the analysis at 1-day intervals. The growth parameters were monitored by OD_730_, and the pinene titers were analyzed on a gas chromatography–mass spectrometer (GC-MS). The error bars represent standard deviations of means (mean ± SD, *n* = 3).

To investigate the influence of genetic modification on the photoautotrophic growth of *Synechococcus*, we compared the photoautotrophic growth rates measured by daily OD_730_ between the WT strain and three transformants of the 7002-PS strain grown in tube photobioreactor for 6 days (Figure 3A). No significant variation was observed among the examined strains under culture conditions of 30°C, 100 μmol photons m^−2^ s^−1^, and 1% (*v*/*v*) CO_2_ aeration. Obviously, metabolic stress caused by the overexpression of transgene and the engineered flux of GPP towards pinene production has no adverse effect on the growth of cyanobacteria grown under the conditions described above.

As the colony PCR results confirmed that the integrated sequence had not entirely replaced the neutral region of pAQ1 between two open-reading frames (Figure 1B), numerous transformants of the 7002-PS strain were supposed to exhibit discrepant production performance. In this work, aeration rates at 0.12 and 0.03 vvm were applied to realize a 72-h and a 6-day period of pinene production, respectively. Two independent transformants of the 7002-PS strain were selected for 72-h pinene production with 10% (*v*/*v*) dodecane and 1% (*v*/*v*) CO_2_ aeration at 0.12 vvm, resulting in the pinene titer at 1.362 ± 0.148 mg L^−1^ and 1.525 ± 0.l45 mg L^−1^ (pinene productivity at 0.110 ± 0.018 mg L^−1^ OD_730_^−1^ and 0.114 ± 0.013 mg L^−1^ OD_730_^−1^), respectively (Figure 3B). The transformant with higher pinene-producing performance, named 7002-PS7, showed relatively stable pinene productivity of 0.246 ± 0.05 mg L^−1^ day^−1^ for a 6-day period of pinene production with 10% (*v*/*v*) dodecane and 1% (*v*/*v*) CO_2_ aeration at 0.03 vvm (Figure 3C). Besides, the *α*/*β* isomer ratios exhibited by the 7002-PS strain were also investigated (Figure 3B). Interestingly, AgPS expressed in *Synechococcus* resulted in a ∼33:67 mixture of *α*- to *β*-pinene, in which a significant increase in *β*-pinene production was realized compared to that produced in *E. coli* (Sarria et al., 2014). We hypothesized that the cellular metabolic environment of *Synechococcus* is suitable for forming the *β*-pinene isomer.

## Conclusion

Pinene is of particular interest due to the excellent potential for its dimer to be aircraft fuel. This study described the construction of pinene-producing strain harboring AgPS-overexpressing cassette in *Synechococcus* and investigated its performance of photoautotrophic growth and pinene production using CO_2_ bubble aeration. Moreover, we determined the concentration of dodecane overlay suitable for collecting volatile pinene under aeration conditions. As a result, we successfully realized the photosynthetic production of pinene directly from CO_2_ with a productivity of up to 1.525 ± 0.l45 mg l^−1^ (*α*/*β* isomer ratios at ∼33:67) after 72 h of cultivation. However, the cultivation condition and the pinene-producing pathway could be further optimized, and the gas-trapping system (Kiyota et al., 2014) is more likely suitable for long-term production. Therefore, there still exists a significant opportunity for higher productivity of pinene in *Synechococcus*.

## Data Availability

The original contributions presented in the study are included in the article/Supplementary Material, further inquiries can be directed to the corresponding authors.
