# Supplementary material for: Photosynthetic Conversion of CO2 Into Pinene Using Engineered Synechococcus sp. PCC 7002
Source: Front Bioeng Biotechnol. 2021 Dec 17;9:779437. doi: 10.3389/fbioe.2021.779437 (PMC8718756; doi:10.3389/fbioe.2021.779437)
Supplement: Supplementary file 1 [file DataSheet1.PDF]

www.gcua.de
created: 20.10.2015

AgPS
sequence derived from Escherichia_coli_K12

Codontable:
http://www.kazusa.or.jp/codon/cgi-bin/showcodon.cgi?species=32049&aa=1&style=N

Ordinate (y-axis): relative adaptiveness <20% <10%

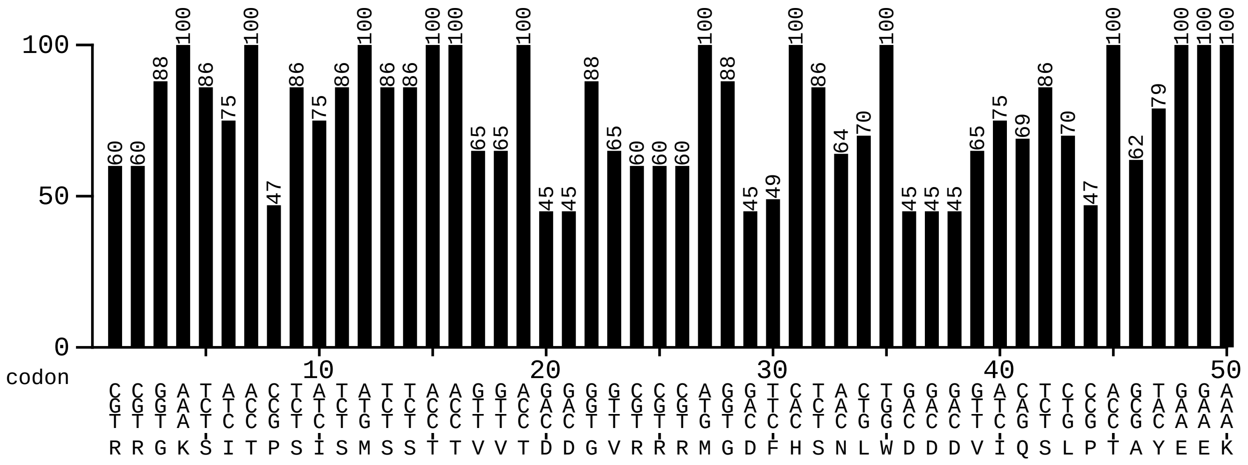

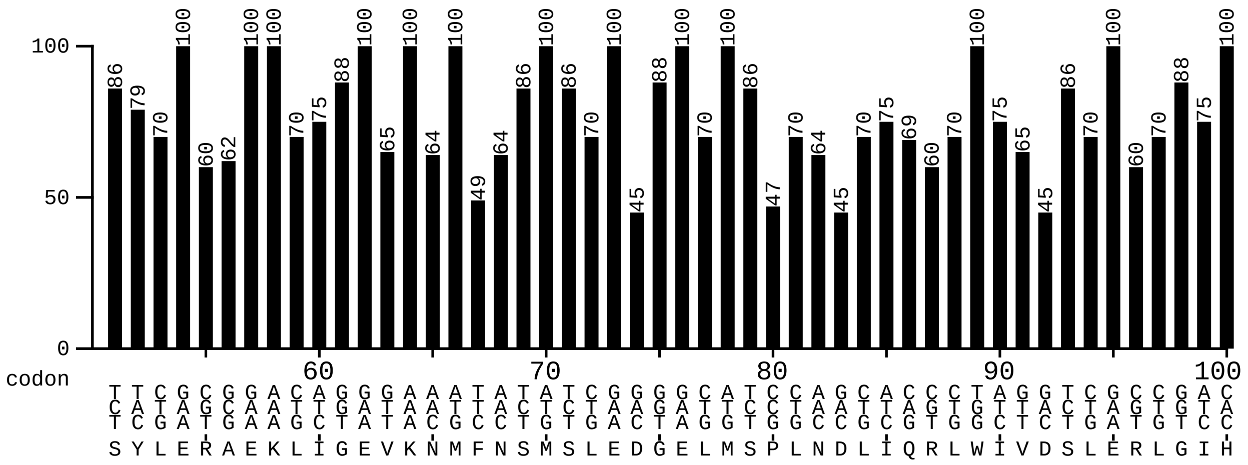

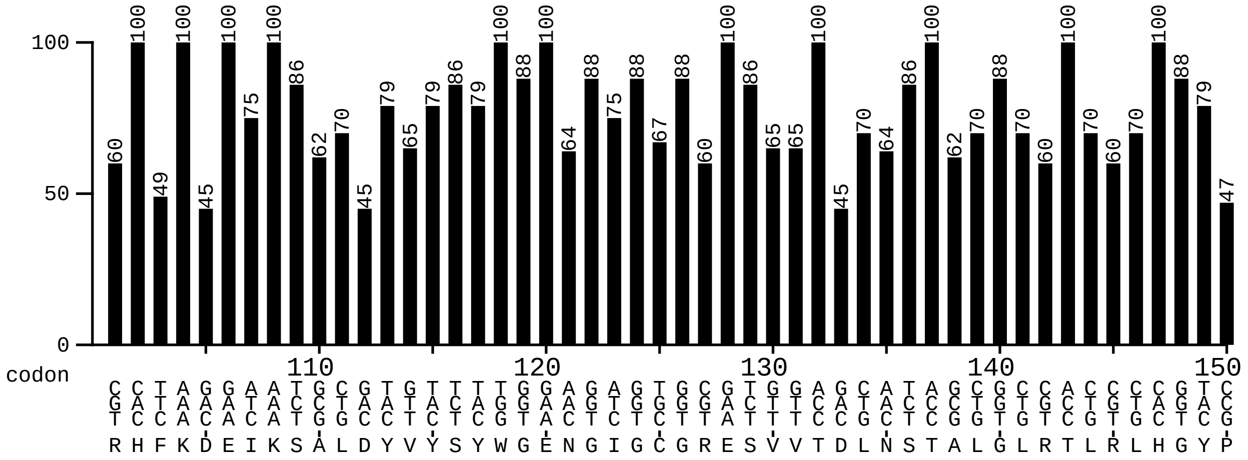

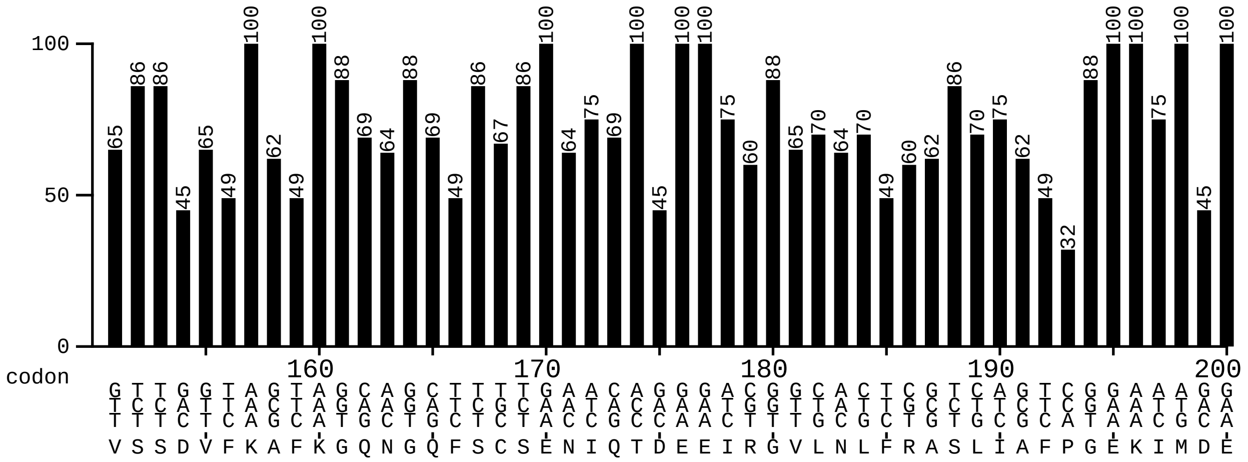
100
50
0
codon
160
170
180
190
200
V S S D V F K A F K G Q N G Q F S C S E N I Q T D E E I R G V L N L F R A S L I A F P G E K I M D E

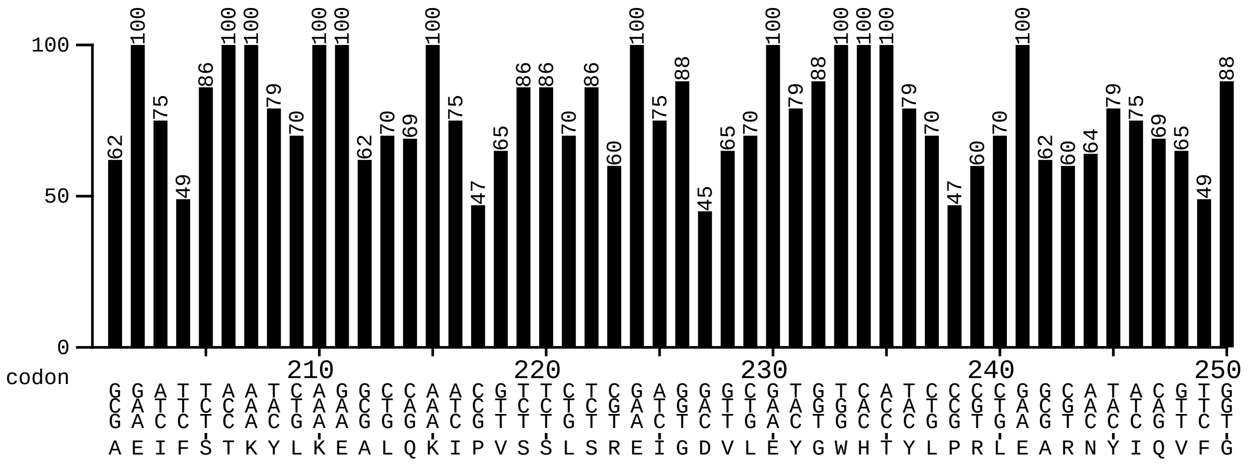
100
50
0
codon
210
220
230
240
250
A E I F S T K Y L K E A L Q K I P V S S L S R E I G D V L E Y G W H T Y L P R L E A R N Y I Q V F G

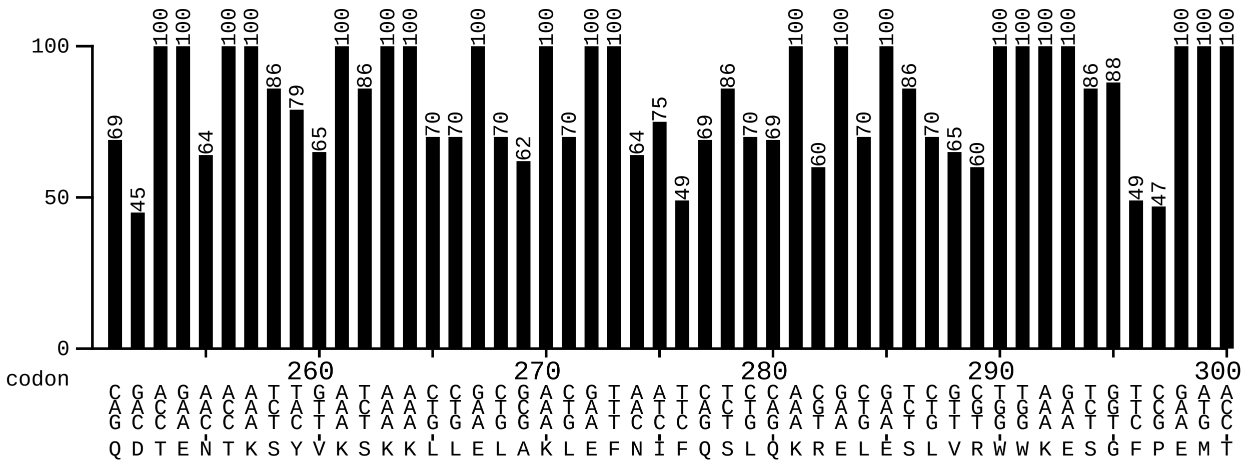
100
50
0
codon
260
270
280
290
300
Q D T E N T K S Y V K S K K L L E L A K L E F N I F Q S L Q K R E L E S L V R W W K E S G F P E M T

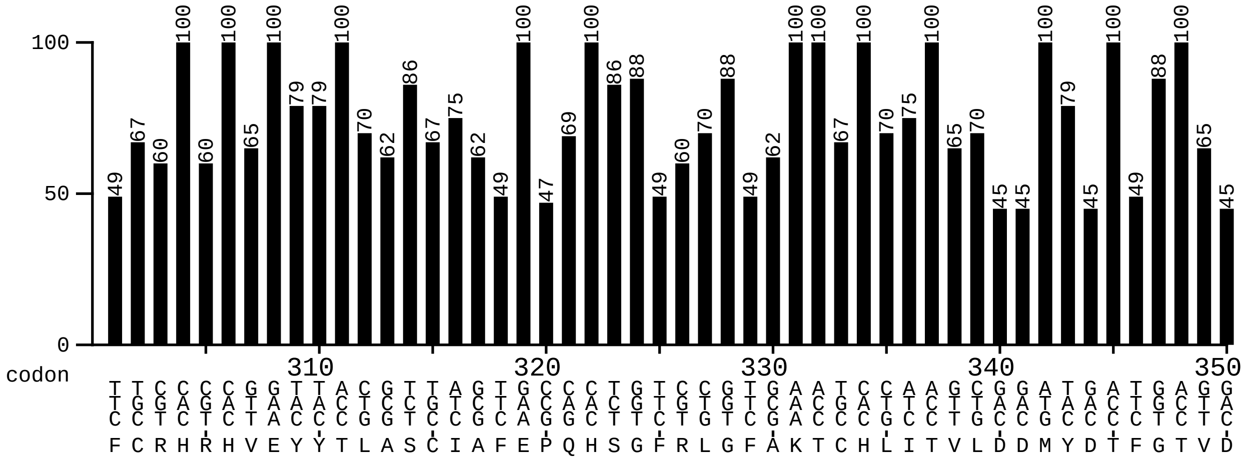
100
50
0
codon
310
320
330
340
350
F C R H R H V E Y Y T L A S C I A F E P Q H S G F R L G F A K T C H L I T V L D D M Y D T F G T V D

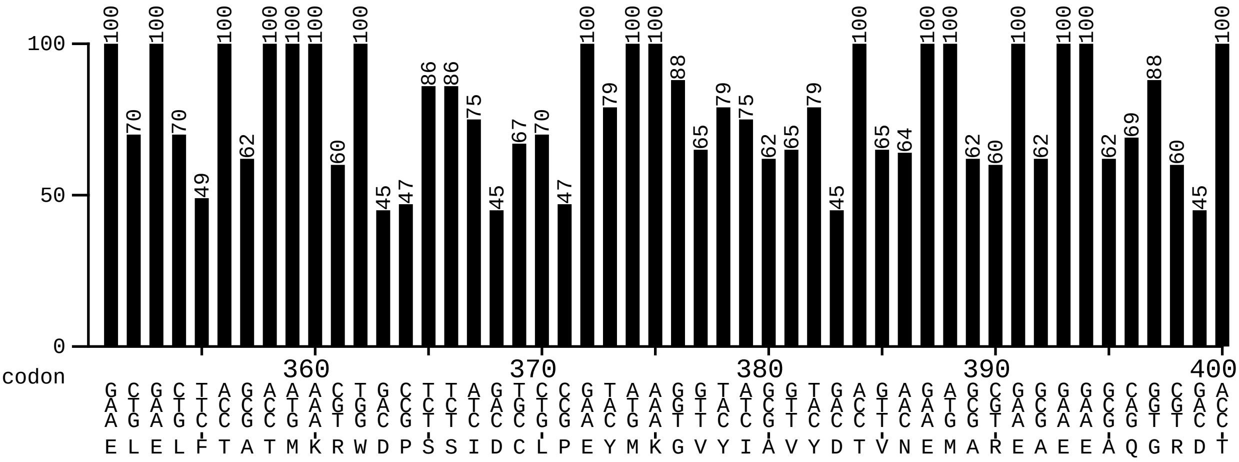
100 70 100 70 49 100 62 100 100 100 60 100 45 47 86 86 75 45 67 70 47 100 79 100 100 88 65 79 75 62 65 79 45 100 65 64 100 100 62 60 100 62 100 100 62 69 88 60 45 100
100
50
0
codon
360 370 380 390 400
G C G C T A G A A A C T G C T T A G T C C G T A A G G T A G G T G A G A G A G C G G G G G C G C G A
A T A T T C C C T A G G A C C C T A G T C A A T A G T A T C T A A C T A A T C G A C A A C A G G A C
A G A G C C G C G A T G C G T T C C C G G A C G A T T C C G T C C C T C A G G T A G A A G G T T C C
E L E L F T A T M K R W D P S S I D C L P E Y M K G V Y I A V Y D T V N E M A R E A E E A Q G R D T

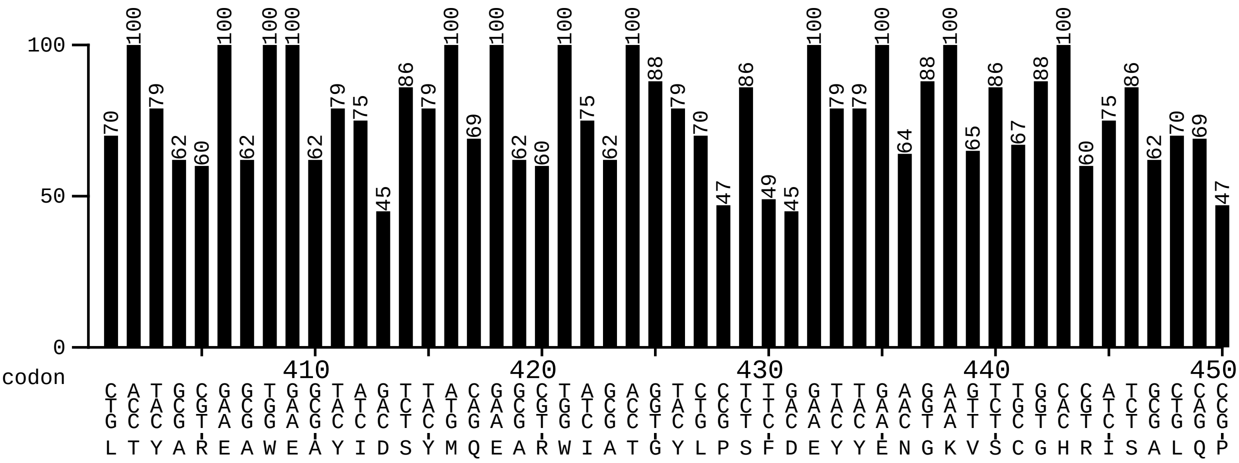
70 100 79 62 60 100 62 100 100 62 79 75 45 86 79 100 69 100 62 60 100 75 62 100 88 79 70 47 86 49 45 100 79 79 100 64 88 100 65 86 67 88 100 60 75 86 62 70 69 47
100
50
0
codon
410 420 430 440 450
C A T G C G G T G G T A G T T A C G G C T A G A G T C C T T G G T T G A G A G T T G C C A T G C C C
T C A C G A C G A C A T A C A T A A C G G T C C G A T C C T A A A A A A G A T C G G A G T C C T A C
G C C G T A G G A G C C C T C G G A G T G C G C T C G G T C C A C C A C T A T T C T C T C T G G G G
L T Y A R E A W E A Y I D S Y M Q E A R W I A T G Y L P S F D E Y Y E N G K V S C G H R I S A L Q P

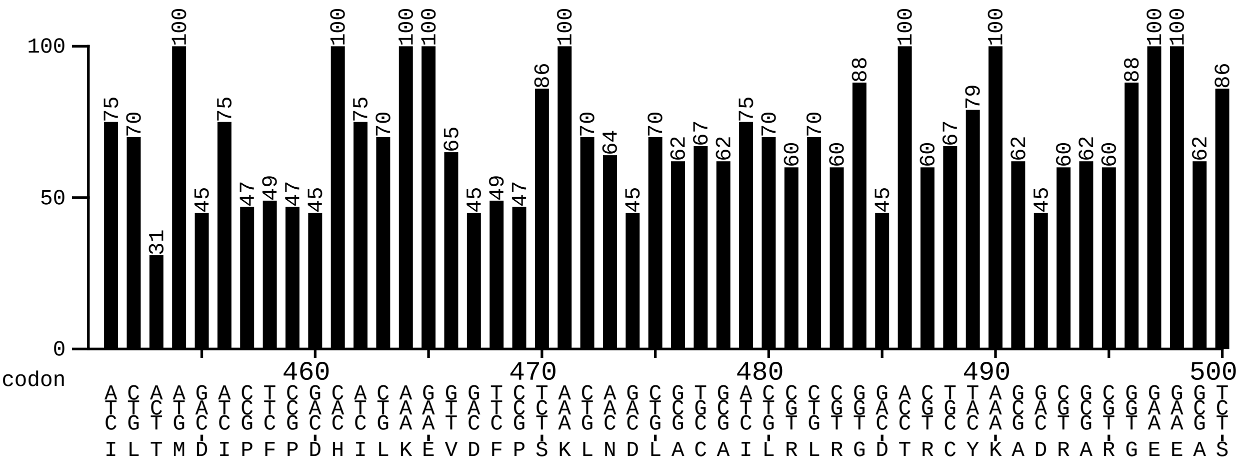
75 70 31 100 45 75 47 49 47 45 100 75 70 100 100 65 45 49 47 86 100 70 64 45 70 62 67 62 75 70 60 70 60 88 45 100 60 67 79 100 62 45 60 62 60 88 100 100 62 86
100
50
0
codon
460 470 480 490 500
A C A A G A C T C G C A C A G G G T C T A C A G C G T G A C C C C G G A C T T A G G C G C G G G G T
T T C T A T C T C A A T T A A T A T C C A T A A T C G C T T G T G G A C G G A A C A G C G G A A G C
C G T G C C G C G C C C G A A T C C G T A G C C G G C G C G T G T T C C T C C A G C T G T T A A G T
I L T M D I P F P D H I L K E V D F P S K L N D L A C A I L R L R G D T R C Y K A D R A R G E E A S

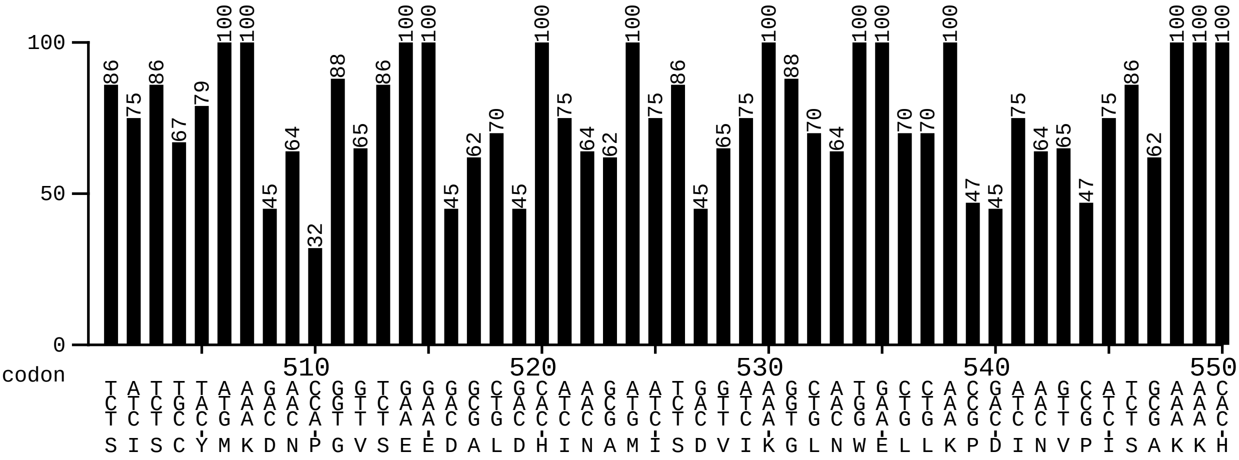
86 75 86 67 79 100 100 45 64 32 88 65 86 100 100 45 62 70 45 100 75 64 62 100 75 86 45 65 75 100 88 70 64 100 100 70 70 100 47 45 75 64 65 47 75 86 62 100 100 100
100
50
0
codon
510 520 530 540 550
T A T T T A A G A C G G T G G G G C G C A A G A A T G G A A G C A T G C C A C G A A G C A T G A A C
C T C G A T A A A C G T C A A A C T A A T A C T T C A T T A G T A G A T T A C A T A T C T C C A A A
T C T C C C G A C C A T T T A A C G G C C C C G G C T C T C A T G C G A G G A G C C C T G C T G A A C
S I S C Y M K D N P G V S E E D A L D H I N A M I S D V I K G L N W E L L K P D I N V P I S A K K H

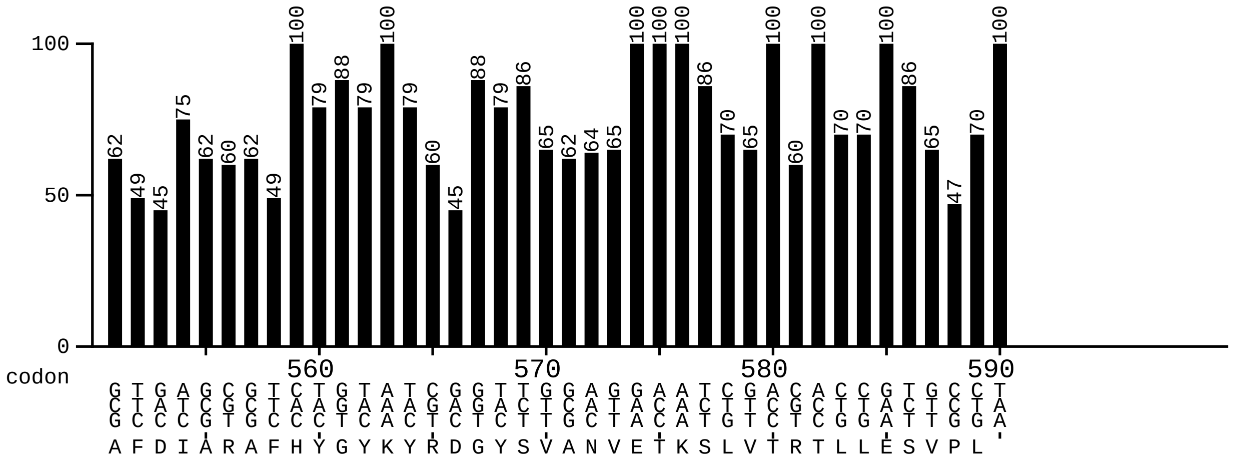
100
50
0
62 49 45 75 62 60 62 49 100 79 88 79 100 79 60 45 88 79 86 65 62 64 65 100 100 100 86 70 65 100 60 100 70 70 100 86 65 47 70 100
codon
560
570
580
590
G T G A G C G T C T G T A T C G G T T G G A G G A A T C G A C A C C G T G C C T
C T A T C G C T A A G A A A G A G A C T C A T A C A C T T C G C T T A C T C T A
G C C C G T G C C C T C A C T C T C T T G C T A C A T G T C T C G G A T T G G A
A F D I A R A F H Y G Y K Y R D G Y S V A N V E T K S L V T R T L L E S V P L
